# Association of advanced lung cancer inflammation index with all-cause and cardiovascular mortality in US patients with rheumatoid arthritis

**DOI:** 10.3389/fnut.2024.1397326

**Published:** 2024-05-30

**Authors:** Zhuang Ma, Shixin Wu, Yitong Guo, Shiyi Ouyang, Ningning Wang

**Affiliations:** ^1^School of Public Health, Guangzhou Medical University, Guangzhou, China; ^2^Department of Health Statistics, School of Public Health, Guangzhou Medical University, Guangzhou, China

**Keywords:** advanced lung cancer inflammation index, all-cause mortality, cardiovascular mortality, rheumatoid arthritis, NHANES

## Abstract

**Introduction:**

As a systemic autoimmune disorder, the prognosis of rheumatoid arthritis (RA) is intricately linked to inflammation. This study aimed to investigate the association between the advanced lung cancer inflammation index (ALI), a comprehensive indicator of inflammation combined with nutritional status, and all-cause and cardiovascular mortality among patients diagnosed with RA.

**Methods:**

The 2,305 RA patients from NHANES (2001–2018) included in the analysis were categorized into three groups according to ALI tertiles. Weighted Kaplan–Meier and multivariate COX regression analyses evaluated the relationship between ALI and mortality. The time-dependent characteristic curve (ROC) was used to assess the prediction accuracy of ALI.

**Results:**

During a median follow-up of 7.92 years, 591 participants died from all causes, including 197 from cardiovascular diseases. Increased ALI was associated with a decreased probability of death. The full COX model revealed lower all-cause mortality hazard risks in the T2 (HR: 0.67, 95%CI: 0.54–0.83) and T3 (HR: 0.47 95%CI: 0.33–0.67, p for tend <0.001) groups compared to T1, and the risk of cardiovascular mortality was also lower in the groups of T2 (HR: 0.47, 95%CI: 0.31–0.70) and T3 (HR: 0.34, 95%CI: 0.19–0.62, p for trend <0.001). Furthermore, the ROC analysis underscored the strong predictive capability of ALI (AUC for 1-year all-cause and cardiovascular mortality were 0.73 and 0.79, respectively).

**Conclusion:**

This cohort study demonstrated the higher accuracy of ALI in predicting mortality in RA patients, highlighting the important clinical value of ALI in risk assessment and prognosis evaluation.

## Introduction

Rheumatoid arthritis (RA), a systemic autoimmune disease, is marked by chronic synovial inflammation and affects approximately 1% of the global population (1). RA inflicts joint damage, and severe pain and significantly impacts the patient’s quality of life, physical function, and mood (2). Furthermore, RA patients are often accompanied by coexisting conditions such as cardiovascular disease and interstitial lung disease, which further contribute to reduced life expectancy (3). Previous studies have indicated elevated mortality rates among RA patients compared to the general population (4–7). Hence, the prognosis of RA patients remains a significant public health concern warranting attention.

Sustained inflammation within RA patients significantly influences the pathogenesis, progression, and prognosis of the disease. Prior research has indicated that C-reactive protein (CRP) can induce the upregulation of nuclear factor-κb ligand (RANKL) in monocytes, initiating osteoclastogenesis and consequent bone resorption (8). Extensive research has consistently demonstrated a direct correlation between heightened levels of inflammation and increased disease activity in RA (9, 10). Furthermore, elevated inflammation levels also correlate with an augmented risk of cardiovascular disease and heightened mortality rates within the RA patient population (11). Nevertheless, it is important to underscore that the inflammatory markers utilized in prior studies are somewhat limited in assessing nutritional status and may not comprehensively capture the inflammatory status of patients. Malnutrition is a common occurrence among RA patients and has been closely associated with an increased risk of all-cause mortality in them (12). There exists a strong correlation between malnutrition and inflammation. Inflammation can trigger muscle proteolysis and impede the repair process, ultimately resulting in muscle loss (13). Furthermore, malnutrition tends to incite a higher inflammatory status (14). Hence, the advanced lung cancer inflammation index (ALI) as a composite marker of nutrition and inflammation, can offer a more comprehensive reflection of the inflammatory status of RA patients compared to other single inflammatory indicators.

ALI was first proposed by Jafri SH as a joint nutrition and inflammation biomarker, including body mass index (BMI), albumin, and neutrophil to lymphocyte ratio (NLR) (15). It was initially employed as a prognostic indicator in various cancers (16–21) and distinguishes itself from previously reported markers or indicators due to nutritional and inflammatory status. Furthermore, the application of ALI has been extended to a few chronic diseases related to nutrition and inflammation (22–25). However, the relationship between ALI and RA remained unknown. Consequently, we first utilized ALI to evaluate its impact on both all-cause mortality and cardiovascular mortality in patients with RA.

The main aim of this study was to investigate the potential link between ALI and both all-cause and cardiovascular mortality in patients with RA, thereby offering a novel perspective and valuable insights into the prognosis and risk stratification of RA patients.

## Materials and methods

This analysis utilized data from a nationally representative sample of the National Health and Nutrition Examination Survey (NHANES), which was conducted by the National Center for Health Statistics (NCHS) to offer a comprehensive evaluation of the health and nutritional status of individuals across various age groups in the US, encompassing both adults and children. NHANES has received approval from the ethics review board of the NCHS, and all participants have provided written informed consent.^1^

### Study design

**Figure 1:**
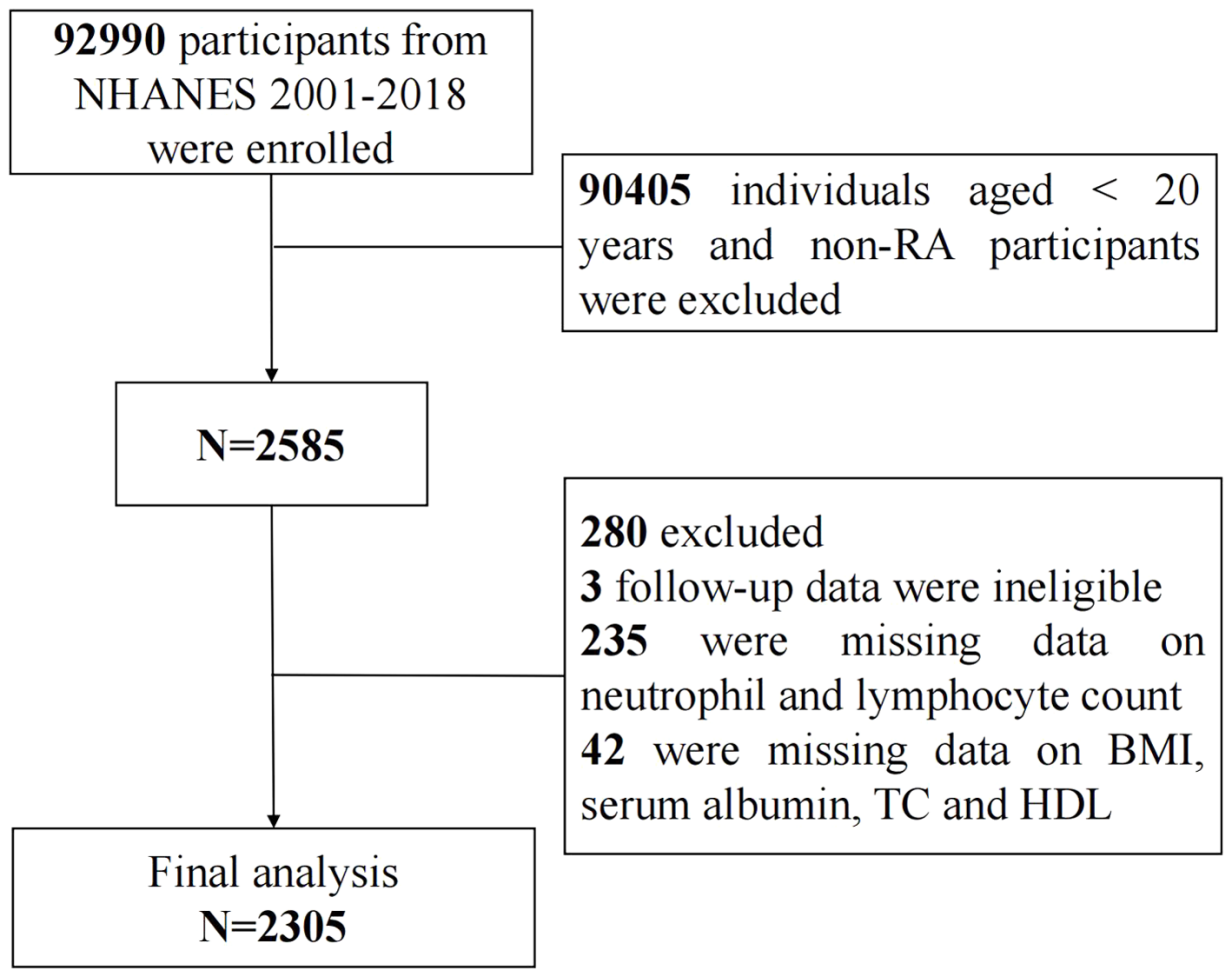
Study design with population inclusion and exclusion.

This cohort study utilized data from nine NHANES cycles spanning from 2001 to 2018, encompassing a total of 92,990 participants. Initially, individuals under the age of 20 and those without rheumatoid arthritis (RA) were excluded (*N* = 90,405). The diagnoses of RA were established through participant self-report, and previous investigations have demonstrated a robust agreement (85%) between self-reported cases and those confirmed clinically (26). Subsequently, 3 participants with ineligible follow-up data, 235 participants with missing data on lymphocyte and neutrophil counts, and 42 participants with absent data on BMI, serum albumin, total cholesterol, and high-density lipoprotein were excluded. Finally, a total of 2,305 participants with RA were included in the follow-up analysis and were categorized into three groups based on ALI tertiles: T1 (ALI ≤ 46.61), T2 (46.61 < ALI ≤ 74.33), and T3 (ALI > 74.33) (Figure 1).

### Assessment of advanced lung cancer inflammation index

Advanced lung cancer inflammation index (ALI) was calculated by the following formula: Alb × BMI/NLR, where Alb represents the serum albumin level (g/dL), BMI is the body mass index of height (kg/m2), and NLR is the ratio of neutrophil count to lymphocyte count (27). Blood cell counts were measured by the Beckman Coulter MAXM instrument, and the DcX800 method was used to measure the albumin concentration.

### Covariates

The covariates considered in the study comprised sex, age, race (Mexican American, other Hispanic, non-Hispanic white, non-Hispanic black, other race), family income poverty ratio (< 1.3, 1.3 to 3.5, ≥ 3.5), BMI (< 25, 25 to 30, ≥ 30). Additionally, smoking status (never, previously, currently) (28), alcohol using status (never, previously, currently), presence of diabetes mellitus (yes, no) (29), hypertension (yes, no) (22), history of cardiovascular disease (CVD) (yes, no) (17), and cancer (yes, no) were included as covariates. Total cholesterol (TC) and high-density lipoprotein (HDL) levels were assessed in specialized laboratories. The duration of RA was determined by calculating the disease duration as the difference between the participant’s current age and the age at which they were initially diagnosed with RA (< 10, ≥ 10 years). A detailed description of covariates can be found in the Supplementary materials.

### Assessment of outcomes

All-cause and cardiovascular mortality were the primary and secondary outcomes, respectively. Mortality data were sourced from the National Death Index (NDI), which is interlinked with the NHANES database.^2^ NDI curated by the NCHS, serves as a database containing mortality data from a diverse range of causes, with a comparatively low probability of error when calculating mortality. The mortality database was recorded through December 31, 2019. Specifically, cardiovascular mortality was identified based on ICD-10 codes (I00–I078) (23).

### Statistical analysis

Statistical analyses were carried out using R software (version 4.3.1; R Foundation for Statistical Computing, Vienna, Austria). A level of *p* < 0.05 (two-tailed) was applied to assess the statistical significance of all analyses.

The statistical analyses conducted in the study adhered to the data analysis protocols outlined by NHANES, taking into consideration its intricate, multistage sampling design. We accounted for the sample’s representativeness by applying weight adjustments post-weight calculation. Continuous variables that followed a normal distribution were reported as mean ± standard deviation (SD), those not conforming to a normal distribution were presented as median (Quartiles), and categorical variables were expressed as counts (Percentages). Demographic characteristics between groups were compared using analysis of variance (ANOVA) and Kruskal-Wallis tests for continuous variables, and the χ^2^ tests for categorical variables.

The univariate COX regression model was used to explore potential factors associated with all-cause and cardiovascular mortality in RA patients. The Kaplan–Meier curve was employed to assess the association between ALI and the survival probability of RA patients across time. Multivariable Cox proportional hazards regression models were utilized to evaluate the association between ALI and all-cause mortality, as well as cardiovascular mortality. Model 1 was unadjusted. Model 2 was adjusted for age, sex, PIR, race, BMI, smoking status, and alcohol use. Model 3 included additional adjustments for TC, HDL, diabetes, hypertension, CVD, cancer, and duration of RA based on Model 2. The weighted median of ALI was applied to all individuals within the subgroup and included as a continuous variable in the full model to assess trends. Restricted cubic spline (RCS) with three knots was implemented in the COX model to examine the potential linear/nonlinear relationship between ALI and all-cause and cardiovascular mortality. RCS adjusted for the same confounding factors as in Model 3. Subsequently, we conducted two sensitivity analyses, the first by excluding individuals who died within 2 years of follow-up, and the second by excluding patients with cancer. We further investigated the relationship between ALI and all-cause as well as cardiovascular mortality by stratified analyses. Interactions were assessed using the Wald test. Lastly, the short-term and long-term predictive value of ALI for all-cause and cardiovascular mortality in patients with RA was assessed using time-dependent receiver operating characteristic (ROC) curves.

## Results

### Baseline demographic characteristics

A total of 2,305 rheumatoid arthritis patients (weighted population 8,188,685) participated in the study analysis, 1,354 (58.7%) were female, and the median age was 58.00 years. According to the tertiles of ALI, the patients were divided into three groups: T1 group (*n* = 768), T2 group (*n* = 769), and T3 group (*n* = 768). Among the three groups, the T3 group exhibited a younger age profile, higher BMI, a higher likelihood of lower household poverty, a greater proportion of non-smokers, and higher levels of TC and serum albumin. The T1 group had a higher probability of being non-Hispanic white, as well as a greater likelihood of having a history of cardiovascular disease and cancer. Furthermore, there were no statistically significant differences observed in gender, alcohol consumption, HDL levels, diabetes, hypertension, and duration of RA among the three groups (Table 1).

**Table 1:** The demographic characteristics of participants (Weighted).

Characteristics	Total	T1 group	T2 group	T3 group	*p*-value
	(*n* = 2,305)	(*n* = 768)	(*n* = 769)	(*n* = 768)	
Weighted population	8,188,685	2,647,642	2,920,345	2,620,698	
Female (%)	1,354 (58.7)	436 (56.8)	443 (57.6)	474 (61.7)	0.244
Age, years	58.00 [48.00, 69.00]	62.00 [50.00, 74.00]	57.00 [46.00, 68.00]	55.00 [46.35, 63.00]	<0.001
BMI, kg/m^2^	29.40 [25.20, 34.20]	26.20 [23.30, 30.58]	29.96 [25.40, 33.85]	31.70 [27.50, 37.55]	<0.001
Race (%)					<0.001
Mexican American	159 (6.9)	51 (6.7)	59 (7.7)	48 (6.2)	
Other Hispanic	118 (5.1)	36 (4.7)	41 (5.3)	39 (5.1)	
Non-Hispanic White	1,526 (66.1)	554 (72.0)	534 (69.5)	434 (56.4)	
Non-Hispanic Black	379 (16.4)	85 (11.0)	102 (13.3)	196 (25.4)	
Other Race	127 (5.5)	43 (5.6)	32 (4.2)	52 (6.8)	
Family poverty income ratio (%)					0.010
< 1.3	591 (25.6)	233 (30.3)	168 (21.8)	193 (25.1)	
1.3 ~ 3.5	922 (40.0)	310 (40.4)	328 (42.6)	281 (36.6)	
≥ 3.5	794 (34.4)	225 (29.3)	274 (35.6)	295 (38.3)	
Alcohol use (%)					0.864
Never	467 (20.2)	168 (21.8)	147 (19.1)	152 (19.7)	
previously	189 (8.2)	61 (8.0)	65 (8.4)	63 (8.2)	
Currently	1,652 (71.6)	540 (70.2)	558 (72.5)	554 (72.0)	
Smoking status (%)					0.007
Never smoker	956 (41.4)	296 (38.4)	295 (38.4)	368 (47.8)	
Former smoker	734 (31.8)	238 (31.0)	253 (32.9)	241 (31.3)	
Current smoker	618 (26.8)	234 (30.5)	221 (28.8)	161 (20.9)	
ALI	59.96 [39.76, 80.12]	32.57 [22.26, 39.28]	60.06 [52.95, 66.64]	94.55 [82.25, 119.46]	<0.001
Lymphocyte, 10^3^/uL	2.00 [1.60, 2.50]	1.50 [1.20, 1.90]	2.00 [1.70, 2.50]	2.40 [2.00, 2.90]	<0.001
Neutrophil, 10^3^/uL	4.20 [3.20, 5.40]	5.10 [4.20, 6.50]	4.20 [3.40, 5.30]	3.20 [2.60, 4.20]	<0.001
HDL, mg/dL	50.00 [41.00, 62.00]	51.00 [42.00, 65.00]	49.00 [40.00, 60.00]	50.00 [42.00, 61.00]	0.145
TC, mg/dL	194.00 [168.00, 222.00]	187.00 [162.30, 216.00]	194.00 [168.00, 223.00]	200.00 [174.00, 223.48]	<0.001
ALB, g/dL	4.10 [3.90, 4.40]	4.10 [3.80, 4.30]	4.10 [3.90, 4.40]	4.20 [4.00, 4.40]	<0.001
Diabetes (%)	535 (23.2)	171 (22.3)	178 (23.2)	186 (24.2)	0.76
Hypertension (%)	1,413 (61.3)	494 (64.3)	455 (59.2)	465 (60.5)	0.282
CVD (%)	547 (23.7)	220 (28.7)	168 (21.9)	160 (20.8)	0.018
Duration of RA ≥ 10 (%)	1,220 (52.9)	407 (53.0)	411 (53.4)	401 (52.2)	0.928
Cancer (%)	361 (15.6)	155 (20.1)	113 (14.7)	92 (11.9)	0.007
Follow-up time, years	7.92 [4.00, 12.42]	7.25 [3.75, 11.67]	8.42 [4.17, 13.00]	8.00 [4.08, 12.71]	0.048

All categorical variables are expressed as counts (percentages); T1 group (ALI ≤ 46.61), T2 group (ALI > 46.61 and ≤ 74.33), and T3 group (ALI > 74.33). All continuous variables are presented as medians [IQR] or as means with standard deviations. BMI, body mass index; ALI: advanced lung cancer inflammation index; HDL, high-density lipoprotein; TC, total cholesterol; ALB, serum albumin; CVD, cardiovascular disease; duration of RA, duration of rheumatoid arthritis.

### Univariate analysis

Univariate Cox regression analysis indicated that age, cancer, hypertension, diabetes, cardiovascular disease (CVD), and RA duration were identified as significant risk factors for both all-cause and cardiovascular mortality in RA patients. Specifically, each one-year increment in age was associated with a 9% rise in the risk of all-cause mortality. Moreover, cancer, hypertension, diabetes, and CVD demonstrated elevated risks of all-cause mortality by 94, 148, 102, and 192%, respectively. Additionally, patients with a duration of RA of more than 10 years exhibited an 86% heightened risk of all-cause mortality. Furthermore, these factors were found to be associated with an increased risk of cardiovascular mortality, as outlined in Table 2.

**Table 2:** Single-factor regression analysis (weighted).

Variables	All-cause mortality	Cardiovascular mortality
	HR (95%CI)	HR (95%CI)
ALI, per 10 U	0.85 (0.81–0.89)^c^	0.79 (0.72–0.86)^c^
Age	1.09 (1.08–1.10)^c^	1.12 (1.09–1.15)^c^
Female	0.84 (0.70–1.01)	0.94 (0.65–1.37)
*Family poverty income ratio*		
1.3 ~ 3.5	1.18 (0.93–1.50)	1.11 (0.74–1.68)
≥ 3.5	0.57 (0.43–0.76)^c^	0.52 (0.32–0.83)^b^
*Race*		
Other Hispanic	0.83 (0.44–1.59)	0.79 (0.30–2.04)
Non-Hispanic White	1.86 (1.30–2.66)^c^	2.01 (1.00–4.04)^a^
Non-Hispanic Black	1.34 (0.96–1.89)	1.60 (0.79–3.26)
Other Race	0.98 (0.54–1.79)	0.84 (0.32–2.26)
*BMI status*		
25 ~ 30	1.05 (0.76–1.44)	1.21 (0.63–2.33)
≥ 30	0.70 (0.53–0.94)^a^	0.94 (0.54–1.61)
*Smoking status*		
Former smoker	1.29 (1.02–1.64)^a^	0.99 (0.63–1.56)
Current smoker	1.07 (0.83–1.37)	0.71 (0.45–1.11)
*Alcohol use*		
Previously	0.72 (0.47–1.03)	0.69 (0.38–1.26)
Currently	0.72 (0.57–0.92)^b^	0.59 (0.39–0.90)^a^
Cancer	1.94 (1.46–2.58)^c^	2.13 (1.35–3.37)^b^
Hypertension	2.48 (1.91–3.24)^c^	2.98 (1.92–4.62)^c^
Diabetes	2.02 (1.61–2.52)^c^	2.90 (2.03–4.14)^c^
CVD	2.92 (2.28–3.75)^c^	4.56 (3.06–6.80)^c^
Duration of RA	1.86 (1.49–2.31)^c^	2.20 (1.46–3.31)^c^
HDL	1.00 (0.99–1.01)	0.99 (0.98–1.00)
TC	1.00 (0.99–1.00)	0.99 (0.99–1.00)^a^

ALI, advanced lung cancer inflammation index; BMI, body mass index; CVD, cardiovascular disease; HDL, high-density lipoprotein; TC, total cholesterol.

a *p* < 0.05.

b *p* < 0.01.

c *p* < 0.001.

### Association of ALI with all-cause and cardiovascular mortality

**Figure 2:**
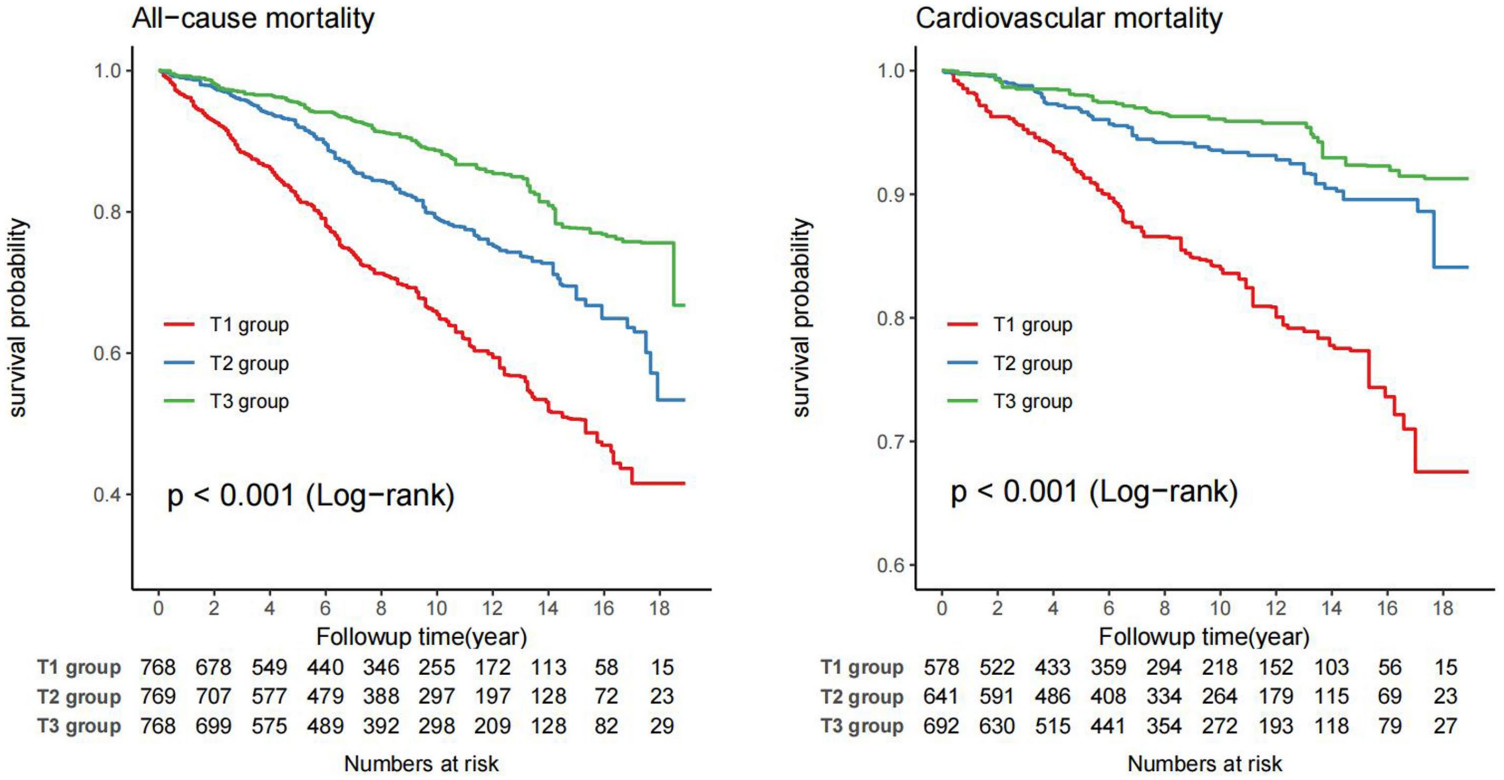
Weighted Kaplan–Meier curves for the association of ALI with all-cause and cardiovascular mortality in RA patients (weighted).

Kaplan–Meier survival curve revealed that the T3 group exhibited a higher long-term survival rate compared to the T1 and T2 groups (*P*-log rank <0.001, Figure 2). Univariate Cox regression analysis demonstrated that both the T2 group (HR: 0.53, 95%CI: 0.43–0.65) and T3 group (HR: 0.30, 95%CI: 0.23–0.41, *P* for trend <0.001) had a lower risk of all-cause mortality compared to the T1 group. The final multivariate Cox proportional hazards model, adjusted for potential confounders, indicated that the T2 group (HR: 0.67, 95%CI: 0.54–0.83) and T3 group (HR: 0.47 95%CI: 0.33–0.67, *P* for tend <0.001) with higher ALI values, had a lower risk of all-cause mortality compared to the T1 group (Table 3).

**Table 3:** The associations of ALI with all-cause and cardiovascular mortality among patients with rheumatoid arthritis were followed up until December 31, 2019 (weighted).

Variables	Case/person	Weighted death	Model1	Model2	Model3
			HR (95%CI)	HR (95%CI)	HR (95%CI)
*All-cause mortality*					
T1 group	297/768	923,059	1(reference)	1(reference)	1(reference)
T2 group	179/769	597,995	0.53 (0.43–0.65)	0.68 (0.55–0.84)	0.67 (0.54–0.83)
T3 group	115/768	314,062	0.30 (0.23–0.41)	0.49 (0.34–0.69)	0.47 (0.33–0.67)
*P* for trend			<0.001	<0.001	<0.001
ALI/per 10 U	591/2305	1,835,116	0.85 (0.81–0.89)	0.91 (0.87–0.95)	0.91 (0.87–0.95)
*Cardiovascular mortality*					
T1 group	107/578	325,284	1(reference)	1(reference)	1(reference)
T2 group	51/641	154,177	0.37 (0.26–0.54)	0.49 (0.34–0.73)	0.47 (0.31–0.70)
T3 group	39/692	95,374	0.24 (0.15–0.37)	0.35 (0.19–0.62)	0.34 (0.19–0.62)
*P* for trend			<0.001	<0.001	<0.001
ALI/per 10 U	197/1911	574,835	0.79 (0.72–0.86)	0.84 (0.77–0.91)	0.85 (0.78–0.92)

HR: hazard ratio, 95% CI: 95% confidence interval. Model 1: Unadjusted. Model 2: Adjusted for age, sex, PIR, race, BMI, smoking status, and alcohol use. Model 3: Further adjusted for HDL, TC, diabetes, hypertension, CVD, cancer, and duration of rheumatoid arthritis.

For the analysis of cardiovascular mortality, a total of 1911 subjects were enrolled after excluding 394 RA patients with non-cardiovascular death. The Kaplan–Meier survival curve also demonstrated that the T3 group had a lower probability of death from cardiovascular compared to the T1 and T2 groups (*P*-log rank <0.001, Figure 2). The adjusted Cox models accounting for confounders indicated that the T2 group (HR: 0.47, 95%CI: 0.31–0.70) and T3 group (HR: 0.34, 95%CI: 0.19–0.62, *P* for trend <0.001) had a lower risk of death from cardiovascular compared to the T1 group (Table 3).

The RCS model illustrated the dose–response relationship between ALI and the risk of all-cause and cardiovascular mortality. It revealed a nonlinear L-shaped negative association between ALI and the risk of all-cause mortality (*P*-overall <0.001 and *P*-nonlinear <0.001). Similarly, a nonlinear L-shaped negative association between ALI and the risk of cardiovascular mortality (*P*-overall <0.001 and *P*-nonlinear <0.001) was observed (Supplementary Figure S1).

### Sensitivity analyses

Two sensitivity analyses produced consistent results. Specifically, after excluding individuals who died within 2 years of follow-up, the comprehensive model showed a reduced risk of all-cause (HR: 0.49, 95%CI: 0.34–0.69, *p* < 0.001, Supplementary Table S1) and cardiovascular (HR: 0.36, 95%CI: 0.19–0.67, *p* < 0.001) mortality in the T3 group with higher ALI. Similarly, after excluding cancer patients, the T3 group with higher ALI continued to demonstrate lower risks of all-cause (HR: 0.47, 95%CI: 0.28–0.73, *p* < 0.001, Supplementary Table S2) and cardiovascular (HR: 0.38, 95%CI: 0.20–0.70, *p* = 0.002) mortality.

### Stratified analyses

**Figure 3:**
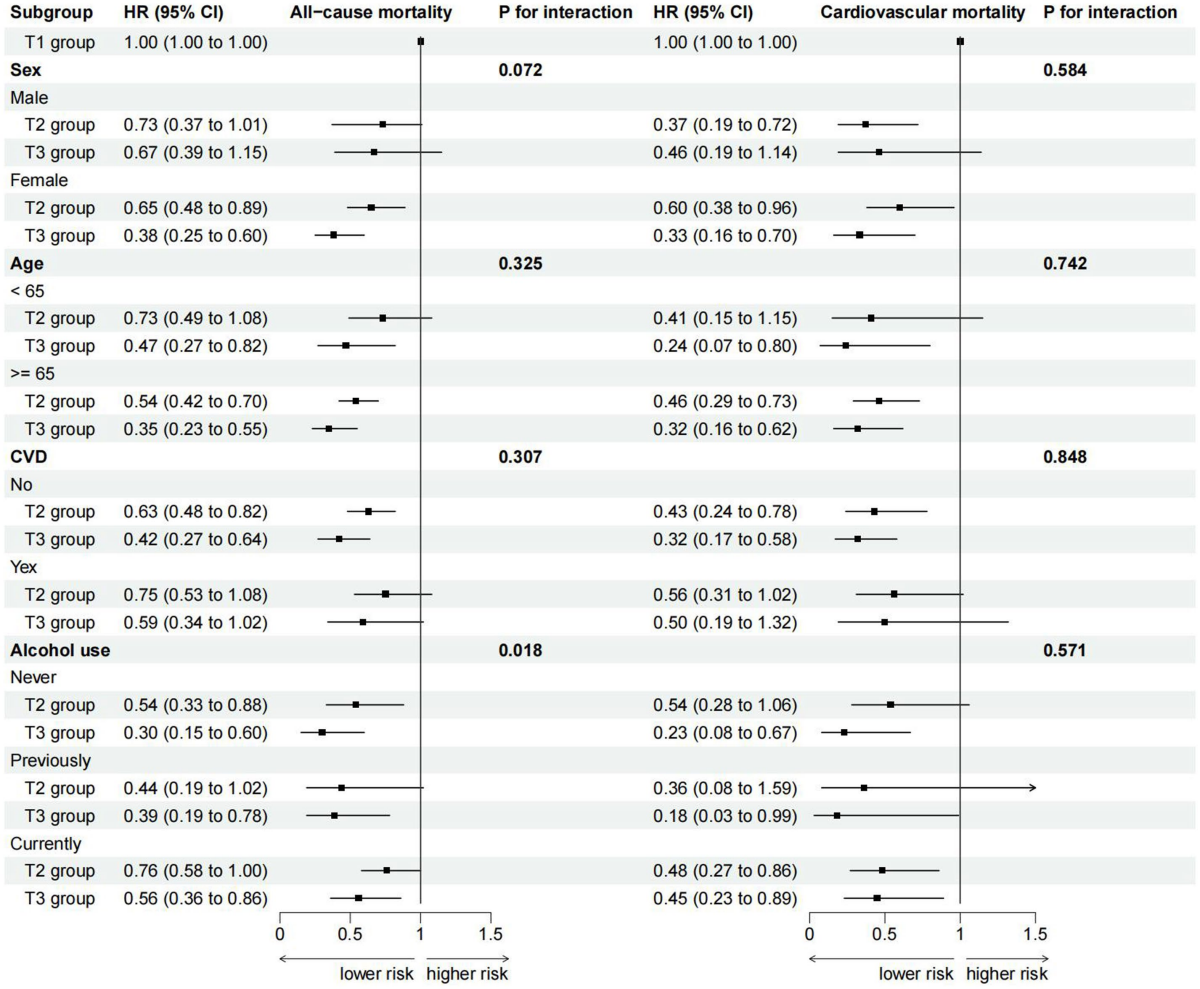
Subgroup analysis for the association of ALI with all-cause and cardiovascular mortality (weighted).

Stratified analyses were conducted among RA patients based on gender, age, history of cardiovascular disease, and alcohol use status to further investigate the relationship between ALI and all-cause mortality, as well as cardiovascular mortality (Figure 3). The results revealed a significant interaction between alcohol use status and ALI (*P* for interaction = 0.018). Specifically, in the subgroup of never-drinkers, a stronger correlation was found between higher ALI levels and a decreased risk of all-cause mortality compared to the subgroup of current drinkers. Similar associations were observed in our subgroup-specific dose–response analyses of ALI and mortality risk (Supplementary Figure S2). Additionally, no interaction was observed in the remaining subgroups, indicating our conclusions had a high level of consistency across different subgroups.

### ROC analysis of evaluating the predictive value of ALI for all-cause and cardiovascular mortality in RA patients

**Figure 4:**
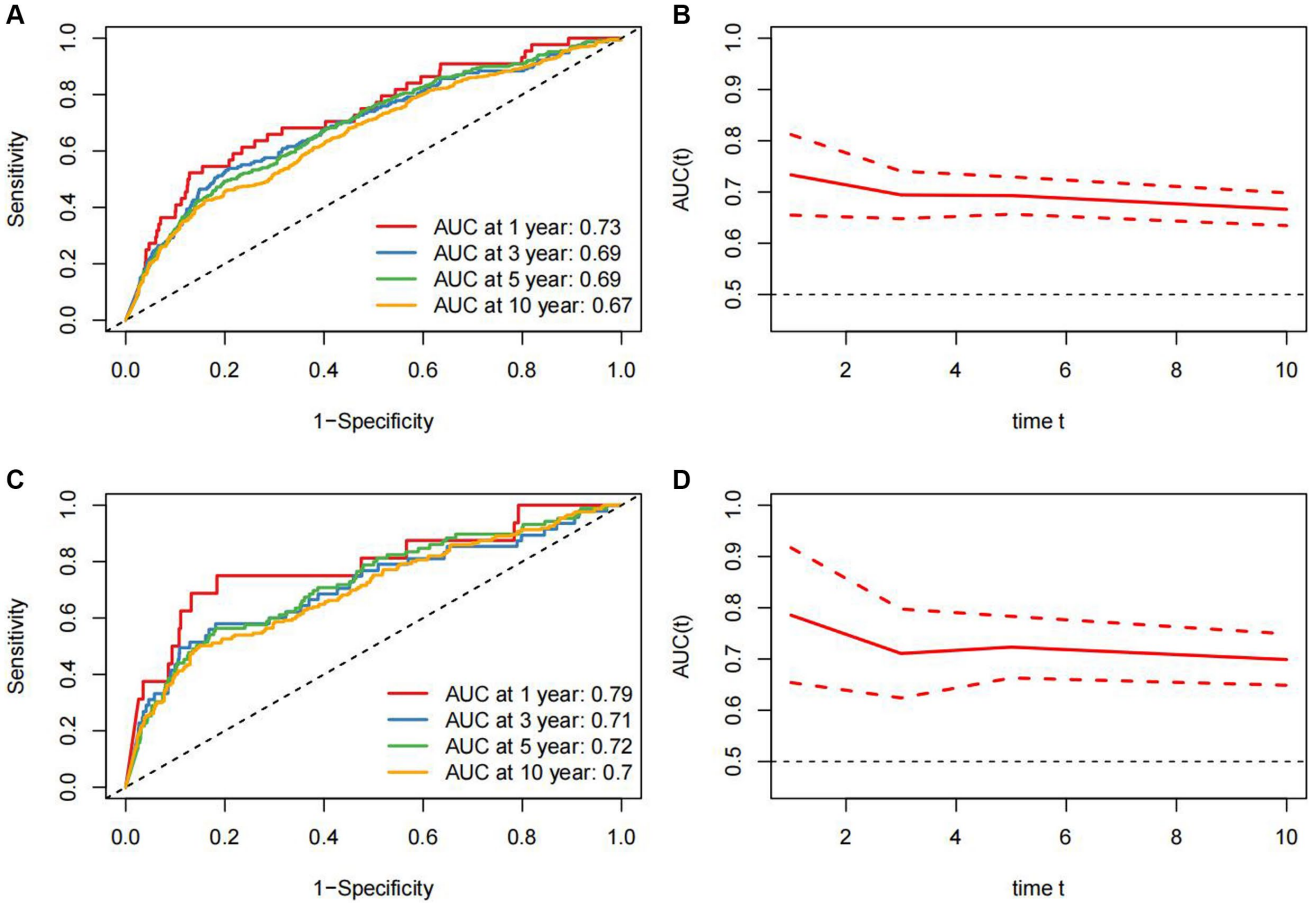
Time-dependent ROC curves and time-dependent AUC values (95% confidence intervals) of ALI for predicting all-cause mortality **(A,B)** and cardiovascular mortality **(C,D)**.

Time-dependent ROC analysis demonstrated that the area under the curve (AUC) of ALI for all-cause death in RA patients was 1-year (AUC: 0.73, 95%CI: 0.65–0.81), 3-year (AUC: 0.69, 95%CI: 0.65–0.74), 5-year (AUC: 0.69, 95%CI: 0.64–0.73), and 10-year (AUC: 0.67, 95%CI: 0.63–0.70), respectively (Figures 4A,B). For cardiovascular mortality, the AUC of ALI was 1-year (AUC: 0.79, 95%CI: 0.65–0.92), 3-year (AUC: 0.71, 95%CI: 0.62–0.80), 5-year (AUC: 0.72, 95%CI: 0.66–0.78), and 10-year (AUC: 0.70, 95%CI: 0.65–0.75), respectively (Figures 4C,D). The findings demonstrated that ALI exhibited a reliable and valid predictive value for both short-term and long-term all-cause and cardiovascular mortality.

## Discussion

In this extensive and nationally representative cohort of US adults, we have identified a robust association between elevated ALI levels and a decreased risk of all-cause and cardiovascular mortality in patients with RA. Kaplan–Meier curves have illustrated a lower probability of death among individuals with higher ALI. Furthermore, RCS analyses have revealed nonlinear inverse correlations between ALI levels and the risks of all-cause and cardiovascular mortality. These findings underscore the practical significance of ALI in risk stratification and prognostic assessment for patients with RA. Furthermore, our ROC analysis has reaffirmed the predictive accuracy of ALI in predicting short-term and long-term survival outcomes in patients with RA, with the AUC for 1-year cardiovascular mortality achieving a value of 0.79.

RA is a chronic immune inflammatory disease, which has been shown to shorten the life span of patients, and the mortality gap between RA patients and the general population is widening (30). The heart is a common organ affected by RA, and previous studies have shown that patients with RA have a higher risk of cardiovascular disease than the general population and are twice as likely to experience sudden cardiovascular death as those without RA (2). In the study by FuH, it was noted that the inflammatory index NLR is linked to the disease activity of RA (31). Subsequently, a researcher has suggested that an elevated NLR is correlated with an increased risk of mortality in RA patients (32). Videm et al. previously proposed inflammation as a mediator of all-cause mortality in RA patients (33). All these results suggest that inflammation has a negative impact on the prognosis of RA patients, aligning with the findings of our study. It is important to highlight that prior studies often focus on individual inflammatory markers, which may not offer a comprehensive assessment of patients’ inflammatory status. Alongside inflammation, nutritional status is a topic of interest. An increasing body of research indicates a potential relationship between nutrition and the onset and prognosis of RA, with a higher prevalence of malnutrition observed in RA patients compared to the general population (34). There exists a complex interplay between malnutrition and inflammation. Khatami (35) emphasized the critical role of malnutrition in the development of systemic inflammation and associated pathology. Additionally, a cohort study reported a heightened risk of all-cause mortality in RA patients who were malnourished (12). Therefore, we contended that assessing the prognosis of RA patients should consider both inflammation and malnutrition concurrently.

ALI was an index combining inflammation and nutrition, comprising BMI, serum albumin, and NLR. ALI was first applied to evaluate the mortality risk of patients with lung cancer (24, 36). Previous research has utilized ALI to assess nutrition and inflammation in individuals with various cancers and chronic diseases (16, 17, 37), highlighting its prognostic value. In a previous investigation by Jafri et al., ALI was shown to enhance the protective factor against all-cause mortality in patients with non-small cell carcinoma (15). A study focusing on heart failure revealed that patients with higher ALI levels had a reduced risk of all-cause mortality and readmission (24). Additionally, evidence suggests that ALI exerts an independent effect on both all-cause and cardiovascular mortality in hypertensive patients (23). It is noteworthy that these studies consistently depict ALI as a protective factor, which is consistent with the direction of our results. No study has yet assessed the association between ALI and mortality rates in RA patients. Our study is the first to demonstrate that among RA patients, an increase in ALI is significantly associated with a decreased risk of all-cause and cardiovascular mortality.

The potential association between elevated ALI levels and decreased all-cause and cardiovascular mortality in patients with RA can be analyzed from three dimensions. Firstly, the immune-inflammatory response represented by NLR plays a crucial role. Elevated NLR levels reflect the functional state of the immune system during chronic inflammation (38). Neutrophils exhibit the capacity to produce TNF-α within inflamed joints, thereby recruiting and stimulating *B* and *T* lymphocytes via the release of CCL18 and B lymphocyte stimulators. Consequently, dysregulation of *B* and *T* cells perpetuates the inflammatory cascade (39, 40). Chronic inflammation and autoimmune processes in RA lead to endothelial dysfunction, promoting the progression of atherosclerosis and increasing the risk of cardiovascular mortality (41, 42), thereby increasing the risk of cardiovascular mortality. Secondly, malnutrition in RA is characterized by a progressive reduction in body protein. Excessive protein catabolism evoked by inflammatory cytokines and disuse atrophy due to functional impairment contributes to this progression (34). Malnutrition further increases the risk of death in RA patients (12). Thirdly, increased BMI is strongly associated with poor prognosis in patients with RA (43). Obesity is considered a systemic inflammatory condition with elevated levels of inflammatory cytokines, including tumor necrosis factor-alpha and interleukin-6, which promote the inflammatory response in RA patients (44). These inflammatory cytokines could promote the inflammatory response of RA patients. Based on the above analysis, we contend that a higher ALI level primarily contributes to a consistent reduction in all-cause and cardiovascular mortality risks for RA patients. Further studies are warranted to elucidate the precise mechanisms underlying this association.

In our subgroup analysis, we found a potential interaction between gender and ALI (*P* for interaction = 0.072), suggesting that there may be gender differences in the relationship between ALI and the overall mortality risk in RA patients. This could be attributed to the significantly higher prevalence of RA in females compared to males (39), with females often experiencing a poorer prognosis (45). Furthermore, research has indicated that sex hormones, such as X, influence the onset of RA (46). Notably, we observed an interaction between alcohol consumption and ALI. The RCS analysis revealed that in the subgroup of current drinkers, the correlation between elevated ALI and decreased risk of mortality was attenuated. Alcohol may activate the complement pathway, and induce the recruitment of neutrophils, thereby influencing NLR levels (47). Furthermore, chronic alcohol consumption can induce oxidative stress in the alveoli, leading to an increase in the production of pro-inflammatory cytokines (48). This mechanism has also been validated in mouse models (49).

Our study possesses several strengths. Firstly, we benefitted from a large sample size, which bolstered the statistical power of our analysis and increased the applicability of our findings to a broader population of RA patients. Furthermore, our sensitivity analyses not only reduced the potential concerns regarding reverse causality but also considered the stronger association of ALI with prognosis in cancer patients, which lends more credibility to our conclusions. Nevertheless, our study is not without limitations. The reliance on self-reported questionnaires for data on RA diagnoses and certain comorbidities may have introduced bias. Moreover, the availability of only baseline data without serial follow-up indicators restricted our ability to accurately evaluate the nutritional and inflammatory status of patients over time. Lastly, since our study focused on RA patients in the United States, further investigation is warranted to ascertain the generalizability of our findings.

## Conclusion

Overall, we have confirmed in this cohort that elevated ALI is significantly linked to a reduced risk of all-cause and cardiovascular mortality in RA patients. The association between ALI and all-cause and cardiovascular mortality followed an L-shaped pattern inversely. Particularly noteworthy is the high level of accuracy of ALI in predicting both all-cause and cardiovascular mortality. These results underscore the potential clinical value of ALI not only as a foundation for risk assessment in clinical practice but also in aiding the evaluation of prognosis in RA patients.

## Data availability statement

The raw data supporting the conclusions of this article will be made available by the authors, without undue reservation.

## Author contributions

ZM: Conceptualization, Software, Writing – original draft. SW: Conceptualization, Writing – original draft. YG: Conceptualization, Writing – original draft. SO: Conceptualization, Software, Writing – original draft. NW: Conceptualization, Funding acquisition, Writing – review & editing.

## Footnotes

^1^ https://wwwn.cdc.gov/nchs/nhanes/Default.aspx

^2^https://www.cdc.gov/nchs/data-linkage/mortality-public.htm
