# Supplementary material for: Association of advanced lung cancer inflammation index with all-cause and cardiovascular mortality in US patients with rheumatoid arthritis
Source: Front Nutr. 2024 May 30;11:1397326. doi: 10.3389/fnut.2024.1397326 (PMC11169605; doi:10.3389/fnut.2024.1397326)
Supplement: Supplementary file 1 [file Data_Sheet_1.docx]

*Supplementary Materials*

1. **Description of covariate definitions**

Age, gender, BMI, race, and family income to poverty ratio (PIR) of participants were obtained from self-report at the time of the interview. The results for the breakdown of covariates were as follows: BMI (< 25, 25 ≤ BMI < 30, ≥ 30 kg/m^2). PIR (<1.3, 1.3 to 3.5, ≥3.5).

Alcohol using status was defined as follows: never (< 12 glass in a lifetime), previously (≥ 12 drinks in 1 year, no drinking in the last year, or no drinking in the last year but ≥ 12 glasses in a lifetime), and currently (≥ 1 glass/day on average in the past 12 months).

Smoking status was categorized as never (< 100 cigarettes in their lifetime), previously (≥ 100 cigarettes in their lifetime, but not currently smoking), and currently (≥ 100 cigarettes in their lifetime, currently smoking occasionally or daily).

Hypertension was defined as meeting any of the following criteria: self-reported diagnosis by a physician or health professional, current use of antihypertensive medication, or average systolic/diastolic blood pressure ≥ 140/90 mmHg during the examination.

Diabetes was considered as a self-report of a physician or medical professional diagnosis, current use of antidiabetic medication, glycosylated hemoglobin (HbA1c) levels ≥ 6.5%, or fasting blood glucose (FBG) levels ≥ 126 mg/dl.

History of cardiovascular disease was defined as a self-report of a previous diagnosis of heart failure, coronary heart disease, angina, heart disease, or stroke.

Cancer was defined as a self-report of ever being diagnosed with cancer or malignancy.

1. **Supplementary Tables and Figures**

**Table S1.** Sensitivity analysis of the relationship between ALI and mortality by excluding individuals who died within 2 years of follow-up; **Table S2.** Sensitivity analysis of the association between ALI and mortality by excluding individuals with cancer; **Figure S1.** RCS with three knots for the nonlinear association of ALI with all-cause (left) and cardiovascular (right) mortality. (weighted); **Figure S2.** Association between ALI and risk of all-cause death in RA patients in subgroups with different alcohol use status.

**2.1 Supplementary Tables**

**Table S1.** Sensitivity analysis of the relationship between ALI and mortality by excluding individuals who died within 2 years of follow-up (Weighted).

| **Variables** | **Case/person** | **Weighted death** | **Model3** | |
| --- | --- | --- | --- | --- |
|  |  |  | **HR (95%CI)** | **P-value** |
| All-cause mortality | | | | |
| T1 group | 241/712 | 738698 | 1(reference) |  |
| T2 group | 161/751 | 532158 | 0.71(0.56-0.91) | 0.006 |
| T3 group | 103/756 | 269702 | 0.49(0.34-0.69) | <0.001 |
| ALI/per 10U | 505/2219 | 1540558 | 0.92(0.88-0.96) | <0.001 |
| Cardiovascular mortality | | | | |
| T1 group | 85/556 | 249948 | 1(reference) |  |
| T2 group | 45/635 | 138722 | 0.53(0.34-0.84) | 0.006 |
| T3 group | 34/687 | 77977 | 0.36(0.19,0.67) | 0.001 |
| ALI/per 10U | 164/1878 | 466648 | 0.87(0.80-0.95) | 0.002 |

Notes: HR: hazard ratio, 95 % CI: 95% confidence interval

Model was adjusted for age, sex, PIR, race, BMI, smoking status, alcohol use, HDL, TC, diabetes, hypertension, CVD, cancer, and course of rheumatoid arthritis.

**Table S2.** Sensitivity analysis of the association between ALI and mortality by excluding individuals with cancer (Weighted).

| **Variables** | **Case/person** | **Weighted death** | **Model3** | |
| --- | --- | --- | --- | --- |
|  |  |  | **HR (95%CI)** | **P-value** |
| All-cause mortality | | | | |
| T1 group | 229/625 | 681304 | 1(reference) |  |
| T2 group | 147/662 | 475140 | 0.74(0.57-0.96) | 0.023 |
| T3 group | 98/682 | 254934 | 0.47(0.33-0.67) | <0.001 |
| ALI/per 10U | 474/1969 | 1411378 | 0.92(0.88-0.96) | <0.001 |
| Cardiovascular mortality | | | | |
| T1 group | 82/478 | 238280 | 1(reference) |  |
| T2 group | 38/553 | 106931 | 0.45(0.28-0.73) | 0.001 |
| T3 group | 38/622 | 92436 | 0.38(0.20-0.70) | 0.002 |
| ALI/per 10U | 158/1653 | 437646 | 0.86(0.79-0.94) | 0.001 |

Notes: HR: hazard ratio, 95 % CI: 95% confidence interval

Model was adjusted for age, sex, PIR, race, BMI, smoking status, alcohol use, HDL, TC, diabetes, hypertension, CVD, cancer, and course of rheumatoid arthritis.

**2.2 Supplementary Figures**


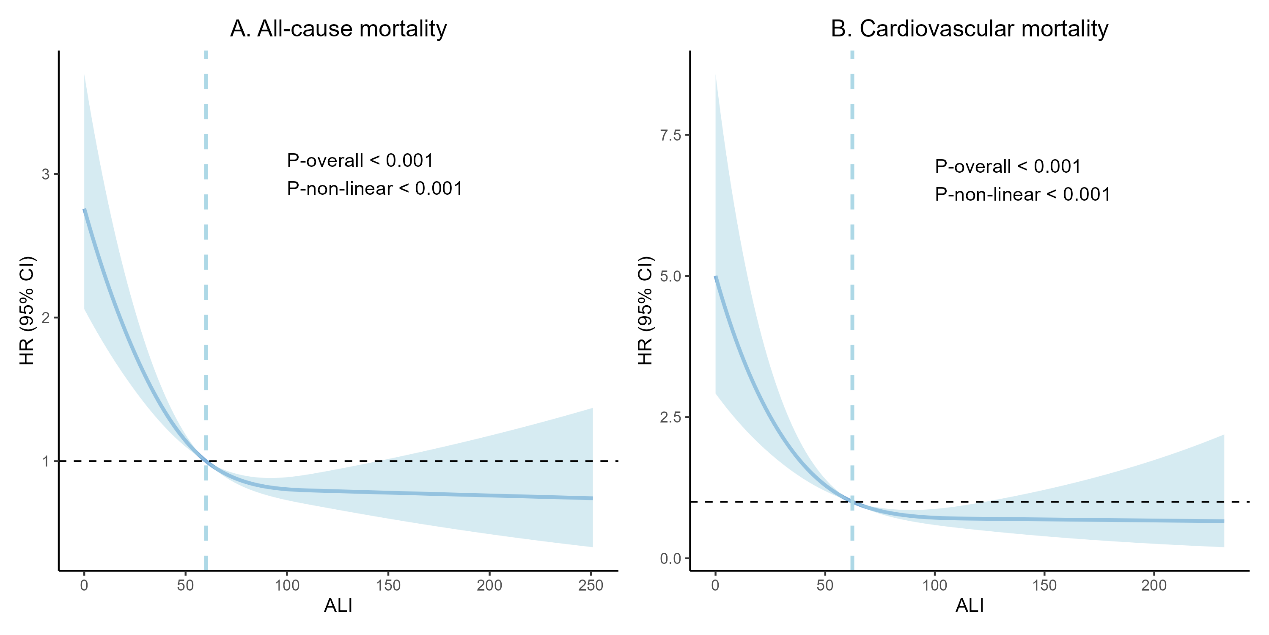


**Figure 1.** RCS with three knots for the nonlinear association of ALI with all-cause and cardiovascular mortality. (weighted)


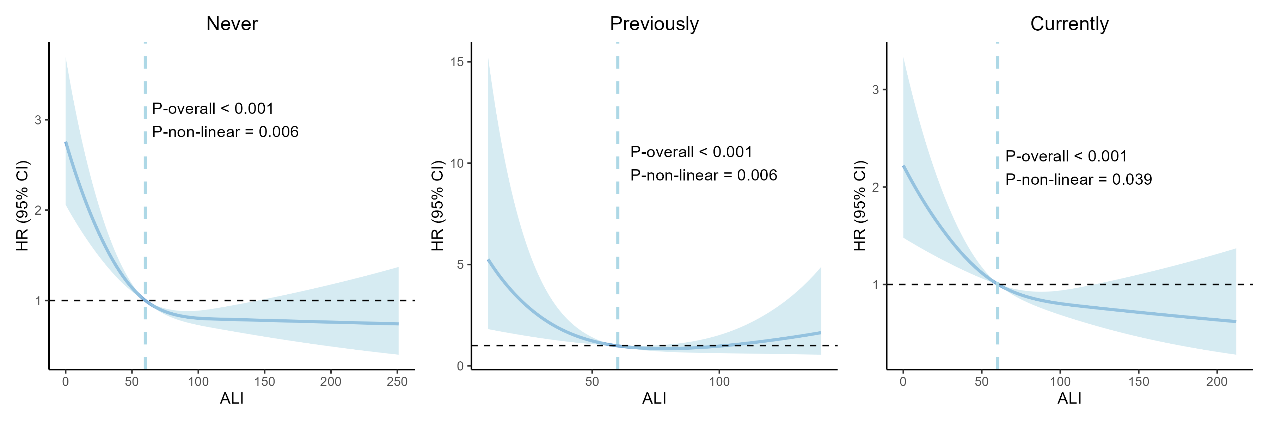


**Figure 2.** Association between ALI and risk of all-cause death in RA patients in subgroups with different alcohol use status.
